# Prion protein localizes at the ciliary base during neural and cardiovascular development, and its depletion affects α-tubulin post-translational modifications

**DOI:** 10.1038/srep17146

**Published:** 2015-12-18

**Authors:** Sophie Halliez, Séverine Martin-Lannerée, Bruno Passet, Julia Hernandez-Rapp, Johan Castille, Céline Urien, Sophie Chat, Hubert Laude, Jean-Luc Vilotte, Sophie Mouillet-Richard, Vincent Béringue

**Affiliations:** 1 INRA (Institut National de la Recherche Agronomique), UR892, Virologie Immunologie Moléculaires, Jouy-en-Josas, France; 2 INSERM UMR-S1124, Université Paris Descartes 75006, Paris, France; 3 INRA, UMR1313, Génétique Animale et Biologie Intégrative, Jouy-en-Josas, France; 4 INRA, Plateforme MIMA2, Jouy-en-Josas, France

## Abstract

Although conversion of the cellular form of the prion protein (PrP^C^) into a misfolded isoform is the underlying cause of prion diseases, understanding PrP^C^ physiological functions has remained challenging. PrP^C^ depletion or overexpression alters the proliferation and differentiation properties of various types of stem and progenitor cells *in vitro* by unknown mechanisms. Such involvement remains uncertain *in vivo* in the absence of any drastic phenotype of mice lacking PrP^C^. Here, we report PrP^C^ enrichment at the base of the primary cilium in stem and progenitor cells from the central nervous system and cardiovascular system of developing mouse embryos. PrP^C^ depletion in a neuroepithelial cell line dramatically altered key cilium-dependent processes, such as Sonic hedgehog signalling and α-tubulin post-translational modifications. These processes were also affected over a limited time window in PrP^C^–ablated embryos. Thus, our study reveals PrP^C^ as a potential actor in the developmental regulation of microtubule dynamics and ciliary functions.

Prions are proteinaceous infectious agents responsible for a broad range of fatal neurodegenerative diseases in animals and humans. They are primarily composed of macromolecular assemblies of PrP^Sc^, a misfolded isoform of the host-encoded PrP^C^. Whether prion toxicity in the central nervous system (CNS) is linked to the generation of toxic PrP^Sc^ (sub)species, to PrP^C^ gain of toxic functions or to the activation of generic toxic pathways^1^ remains a fiercely debated issue, substantially due to the elusive physiological functions of PrP^C^^2^. Unravelling the functions of PrP^C^ may have a broader significance given its increasingly apparent role in mediating toxic signalling associated with more common neurodegenerative diseases such as Alzheimer’s disease^3^. Moreover, PrP^C^ possesses highly conserved primary and tertiary structures among mammals, and the presence of genes homologous to *Prnp* (the gene encoding PrP^C^) in birds, reptiles, amphibians and fish lends support for evolutionarily conserved functions^2^.

PrP^C^ is a ubiquitously expressed, glycosylphosphatidylinositol-anchored cell surface sialoglycoprotein that is present in specific membrane domains termed lipid rafts, which are critical to the biology of the cell^4^. PrP^C^ is involved in a variety of cytoprotective cellular functions and signal transduction pathways^5^. In the last decade, studies of cell systems depleted in or overexpressing PrP^C^ have linked PrP^C^ to the self-renewal of haematopoietic^6^ and embryonic^7^,^8^ stem cells and to the proliferation and/or differentiation of embryonic stem cells^9^ and neural progenitors^10^. However, our knowledge of the underlying cellular mechanisms remains limited. Further complicating the issue is the absence of any drastic phenotype in adult mice upon embryonic^11^ or post-natal^12^ inactivation of *Prnp*, although transient alterations in cell differentiation were later identified in these animals^10^,^13^. The upregulation of *Prnp* expression can be detected as early as embryonic day (E) 6.5 in extra-embryonic regions and E8 in the embryo proper^14^,^15^,^16^, thus supporting a developmental role for PrP^C^. However, the embryonic expression pattern of PrP^C^ is poorly documented; its presence in neural progenitors can be questioned, with some studies reporting initial expression during differentiation^10^,^16^.

To characterize how the PrP^C^ expression pattern is specified relative to stem/progenitor cell fate *in vivo*, we examined the spatiotemporal distribution of PrP^C^ in early developing wild-type mouse embryos. From E8.25 onwards, we found that PrP^C^ was enriched at the base of the primary cilium in stem cells and progenitors of the CNS, heart and forming blood vessels. In the mouse developing neural tube and even more markedly in a neuroepithelial cell line displaying neuroectodermal progenitor hallmarks, the depletion of PrP^C^ altered key cilium-dependent processes and pathways, including α-tubulin post-translational modifications (PTMs) and the Sonic hedgehog signalling pathway. Collectively, these data reveal a new link between PrP^C^ and microtubule dynamics as well as primary cilium functions during development.

## Results

### PrP^C^ is expressed in stem and progenitor cells from the developing central nervous system and cardiovascular system

**Figure 1:**
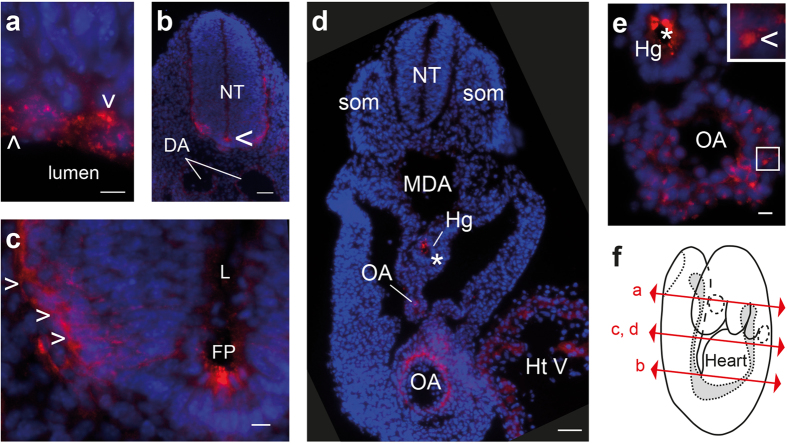
PrP^C^ expression pattern in the nervous and cardiovascular systems from early developing mouse embryos. PrP^C^ (red) and nuclear marker 4’,6-diamidino-2-phenylindole (DAPI, blue) staining of transverse sections from FVB/N mouse embryos at E9 in the developing nervous (**a–c**) and cardiovascular systems (**d,e**). Section plans are indicated (**f**). PrP^C^ was observed at the level of the apical face of the neuroepithelium in the head region (presumptive telencephalon; **a**), in the neural tube (NT) in the trunk region (**b**), and particularly in the floor plate (FP, **c**). The arrowhead indicates PrP^C^-positive differentiating cells in the mantle zone (**c**). Low-exposure acquisition of two juxtaposed embryo sections from the proximal and caudal trunk regions showed strong PrP^C^ expression in the omphalomesenteric artery (OA) and in the embryonic heart ventricle (HtV) (**d**). Higher magnification of the omphalomesenteric artery (OA) (**e**). The asterisks indicate artefact signals found in FVB/N and in *Prnp*^*−/−*^ mice (supplemental Figure S1), likely due to nonspecific Fc binding to maternal blood, and the arrowheads highlight specific punctuate or patchy PrP^C^ signals. Scale bar: 10 μm, except in (**b**,**d**) 50 μm. DA: dorsal aorta, Hg: hindgut, L: lumen, MDA: midline dorsal aorta, som: somite.

We examined the temporal and spatial PrP^C^ expression pattern during mouse embryonic development by immunofluorescence microscopy after labelling with the anti-PrP antibody Sha31, which exhibits high affinity for both full-length and truncated isoforms of PrP^17^. *Prnp*^*−/−*^ embryos were used as negative controls to check for any non-specific staining (e.g., Fig. S1g,h). Representative images are shown in Figs 1 and S1.

At E8.25, in the embryo proper, PrP^C^ was readily detected in the developing forebrain (optic pit region) as puncta at the apical face of the neuroepithelium (Fig. S1a) and as patches in endothelial cells of the forming dorsal aorta (Fig. S1b). PrP^C^ expression was stronger in the developing heart, and the signal appeared mostly as a patchy pattern (Fig. S1c). More caudally, PrP^C^ expression was observed neither in the neural groove (Fig. S1b) nor in the neural tube (Fig. S1e). PrP^C^ patches were also detected in extra-embryonic regions such as the splanchnic mesoderm of the yolk sac (Fig. S1f). This region contains blood islands where primitive haematopoiesis^18^ and vasculogenesis^19^ occur.

At E9, PrP^C^ expression was greater in the CNS (Figs 1a–c and S1j) and cardiovascular system (Figs 1d–f and S1k–m). PrP^C^ puncta were abundant in the entire presumptive brain at the ventricular surface of the neuroepithelium (Fig. 1a) and in the floor plate in the trunk region, an essential organizing centre for the developing CNS (Fig. 1b,c). A fainter signal was observed at the apical face in other regions of the neural tube (Fig. S1j). PrP^C^ patches and puncta together with membrane staining were observed in the mantle zone, where the first differentiating cells migrate (Fig. 1c). In the cardiovascular system, the strongest PrP^C^ signals were observed in the omphalomesenteric artery (Fig. 1d–e), a site of definitive haematopoiesis^20^, and in the developing heart (Figs 1d and S1k–l). The venous pole of the heart, which corresponds at this early developmental stage to conduits draining to the inlet of the heart tube^21^, displayed PrP^C^ membrane staining and intense puncta (Fig. S1k–l). In the omphalomesenteric artery, patches were present on endothelial cells (Fig. 1e). Other vessels, such as the dorsal aorta, also expressed PrP^C^ in the endothelium, albeit at lower levels (Fig. S1m and data not shown). PrP^C^ also remained expressed in the extra-embryonic yolk sac at E9 (not shown).

There was no drastic change in the regional expression pattern of PrP^C^ between E9 and E10.5. Nonetheless, more intense labelling was observed in the neural tube mantle zone as the number of differentiating cells increased (data not shown).

Collectively, these data indicate that PrP^C^ is expressed during early embryonic stages in neuroepithelial cells, in cells from a stem cell niche associated with definitive haematopoiesis and in progenitors of the heart and blood vessels.

### PrP^C^ localizes at the base of the primary cilium

**Figure 2:**
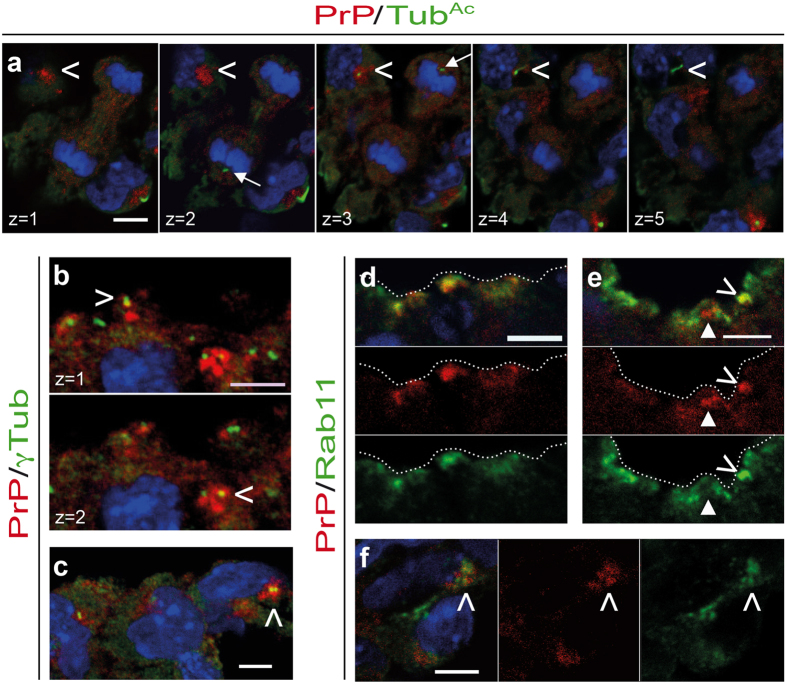
PrP^C^ localization at the base of the primary cilium in progenitor cells of the developing nervous system and in the omphalomesenteric artery. Confocal microscopy imaging of transverse sections of FVB/N mouse embryos at E9-9.5 co-stained for PrP (red) and (green) acetylated tubulin (Tub^Ac^) (**a**), γ-tubulin (γTub) (**b**,**c**) or Rab11 (**d–f**). Nuclei are stained with DAPI (blue). Merged confocal images or individual channels are shown. Z-series (**a**,**b**) or single optical sections (**c–f**) are presented. Co-localization of PrP^C^ with Tub^Ac^ at the level of the omphalomesenteric artery (**a**). The arrowhead highlights PrP^C^-positive punctiform structures at z = 1. Along the z-axis, a primary cilium, as probed by Tub^Ac^, progressively appears (from z = 3), while the PrP^C^ signal progressively disappears. White arrows point to the centrosomes of a mitotic cell and highlight the absence of detectable PrP^C^. Co-localization of PrP^C^ with γTub (arrowheads) at the apical face of the neuroepithelium (forebrain; **b**) and in cells from the omphalomesenteric artery (**c**). Co-localization (arrowheads) or co-regionalization (plain arrowheads) between PrP^C^ and Rab11 at the apical face of the neuroepithelium in the forebrain (**d**), at the floor plate of the neural tube in the trunk region (**e**), and at the omphalomesenteric artery (**f**). The dotted lines delimit the lumen. Scale bar: 5 μm.

Using confocal imaging, we next sought to define the subcellular localization of the PrP^C^ patches and puncta. The primary cilium is a microtubule-based versatile organelle composed of an axoneme extending from the mother centriole-derived basal body and surrounded by a ciliary membrane. Sections of E9-E9.5 FVB/N embryos were co-labelled with anti-PrP and either anti-acetylated tubulin (Tub^Ac^, Figs 2a, S2a–c and S3) or anti-γ-tubulin (γ-tub, Figs 2b–c, S2d–f and S3) antibodies as ciliary axoneme and centriole markers^22^, respectively. In endothelial cells from the omphalomesenteric artery (Fig. 2a,c, Fig. S3), in capillaries of the yolk sac (Fig. S2a), in cells from the venous pole (Fig. S2b,d) and in cardiomyocytes (Fig. S2e), PrP^C^ patches and puncta were identified as punctiform structures at the ciliary base. PrP^C^ was not detected along the axoneme. Quantitative analyses of the z-series showed that 90 ± 9% and 91 ± 10% of Tub^Ac^-positive primary cilia had positive PrP^C^ signals at their base in the omphalomesenteric artery and capillaries of the yolk sac. Conversely 85 ± 8% and 83 ± 12% of PrP^C^ patches localized at the ciliary base (*n* = 7 and *n* = 8 z-series analysed from *n* = 3 embryos, respectively). Tub^Ac^ staining was not restricted spatially to the cilium in neuroepithelial cells, but the PrP^C^ and Tub^Ac^ co-labelling was compatible with the presence of PrP^C^ at the ciliary base (Fig. S2c), as also suggested by the close association or co-localization of PrP^C^ with γ-tub (Fig. 2b, Fig. S3). Quantitatively, 82 ± 6% of γ-tub signals in the floor plate co-localized with PrP^C^ signals. Conversely 98 ± 4% of PrP^C^ puncta co-localized with γ-tub signals (*n* = 5 z-series analysed from *n* = 3 embryos).

In differentiated cells from the mantle region of the neural tube, PrP^C^ still co-localized with γ-tub, despite exhibiting wider expression (Fig. S2f). Importantly, the PrP^C^ punctiform structures never co-localized with Tub^Ac^ (Fig. 2a) or γ-tub (Fig. S2d) positive centrioles from the mitotic spindles, further reinforcing the view that at this developmental stage, PrP^C^ was specifically enriched at the base of the primary cilium in a wide range of stem cells (or cells associated with stem cell niches) and progenitors.

Considering the localization of PrP^C^ at the ciliary base and the intense trafficking of Golgi and endocytic vesicles there to insure proper ciliogenesis^23^, we next examined whether PrP^C^ would co-localize with the recycling endosome marker Rab11. Rab11 is enriched at the ciliary base and is required for primary ciliogenesis^24^. A large proportion of PrP^C^ co-localized with Rab11 or was present in its immediate vicinity in neuroepithelial cells (Fig. 2d–e), in the omphalomesenteric artery (Fig. 2f), in the yolk sac (Fig. S2g) and in the developing heart (Fig. S2h–i). In marked contrast, the most prominent PrP^C^-positive structures in differentiating cells from the mantle zone of the neural tube did not associate with Rab11 (Fig. S2j). Altogether, these data suggest that PrP^C^ is specifically enriched in the pericentriolar recycling compartment of the primary cilium from progenitors and cells associated with stem cell niches of the developing central nervous and cardiovascular systems.

### PrP^C^ depletion affects Sonic hedgehog signalling

**Figure 3:**
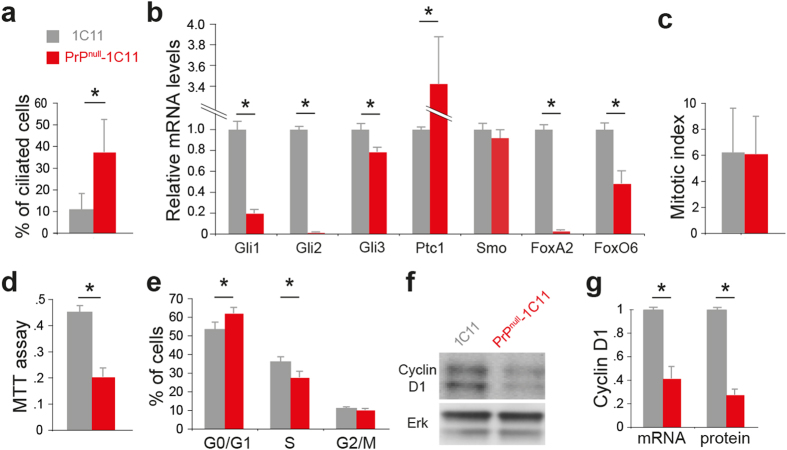
Primary cilium biology in PrP^C^-depleted 1C11 cells. 1C11 and PrP^C^-depleted cells (PrP^null^-1C11) were compared with regard to the percentage of ciliary cells (**a**, *n* = 30 fields of view (~700 cells)). qPCR results showing the expression of Shh-related transcription factors, the Shh receptor Patched 1 (Ptc1) and Smoothened signal transducer transmembrane protein (Smo) are presented ((**b**), (*n* = 3)). The mitotic index ((**c**), *n* = 19 fields of view analysed per cell type, analysis on 3000–6000 cells), cell proliferation ((**d**), *n* = 3 MTT), proportion of cells in the different phases of the cell cycle ((**e**), *n* = 3), protein and transcriptional levels of Cyclin D1 ((**f,g**) and an immunoblot using Erk to normalize the protein levels ((**f**) *n* = *3*) are shown. (*p < 0.05, Student test (**a**) or Mann-Whitney test (**b–g**)). The gels shown in panel (**f**) have been cropped for clarity and conciseness purposes, and have been run under the same experimental conditions; the original blots are shown in supplemental Figure S8a.

To examine the potential impact of PrP^C^ depletion on primary cilium biology in stem cells/progenitors, we first used a neuroepithelial cell line that displays neuroectodermal progenitor hallmarks and has been instrumental in identifying PrP^C^-dependent signalling cascades^25^,^26^. PrP^C^ can be constitutively knocked down in 1C11 cells by an anti-*Prnp* shRNA, resulting in a >95% decrease in PrP^C^ levels^27^. These cells are referred to as PrP^null^-1C11 cells. Tub^Ac^ labelling of 1C11 versus PrP^null^-1C11 cells revealed that the proportion of ciliated cells markedly increased by 3-fold (Fig. 3a), suggesting altered primary cilium turnover^22^ in the absence of PrP^C^. Primary cilia are essential for the transduction of the Sonic hedgehog (Shh) morphogen signal, a key signal in the developmental regulation pathways of multicellular organisms^28^. Cilium abnormalities are usually coupled to Shh signalling pathway alterations^22^,^29^, affecting the expression of effectors of the Shh pathway such as the Gli transcription factors and the Patched1 (Ptc1) Shh receptor. Our qPCR analyses of mRNA isolated from 1C11 and PrP^null^-1C11 cells revealed that Gli1 and Gli2 expression levels were downregulated by >5-fold and 95-fold, respectively, in the absence of PrP^C^, whereas Gli3 levels were reduced by 20% (Fig. 3b). By contrast, Ptc1 transcripts were upregulated by >3-fold in the absence of PrP^C^ (Fig. 3b). The Shh-induced transcription factor and Gli2-regulated FoxA2^30^ showed nearly total transcriptional abolition (Fig. 3b). The increase in Ptc1 transcripts in PrP^null^-1C11 cells, which stands in apparent contradiction to Gli1 downregulation^31^, may be readily explained by the absence of FoxA2, which negatively regulates the transcription of Ptc1^32^. Additionally, mRNA expression of the transcription factor FoxO6, a recently identified Gli target^31^, was significantly lowered by 50% in PrP^null^-1C11 cells (Fig. 3b). Finally, PrP^C^ depletion had no impact on the mRNA levels of Smoothened (Smo, Fig. 3b), a Shh pathway signal transducer, arguing against a global, unspecific effect of PrP^C^ depletion on the Shh pathway.

As Shh signalling controls the cycling of neural precursor cells^33^, we next investigated whether this process was impacted. No significant difference in the mitotic index was observed between 1C11 and PrP^null^-1C11 cells (Fig. 3c). However, the mitotic index may not correlate with the proliferation rate as the duration of the M phase could vary between the two cell populations. We thus refined our cell proliferation studies by using MTT cell proliferation assay and cell cycle analyses^34^. PrP^null^-1C11 cell proliferation was significantly decreased by >50%, as quantified by the MTT assay (Fig. 3d), and the proportion of cells in the G0/G1 and S phases was slightly but significantly increased (Fig. 3e). In line with the lack of difference in the mitotic index between 1C11 cells and PrP^null^-1C11 cells, the proportion of PrP^null^-1C11 cells in G2/M phases was not affected (Fig. 3e). In addition, qPCR and immunoblot analyses showed decreased transcriptional and protein levels of Cyclin D1 (Fig. 3f,g), a regulator of the entry into S phase^35^. Collectively, these data suggest that PrP^C^ depletion affects 1C11 cell cycle regulation.

**Figure 4:**
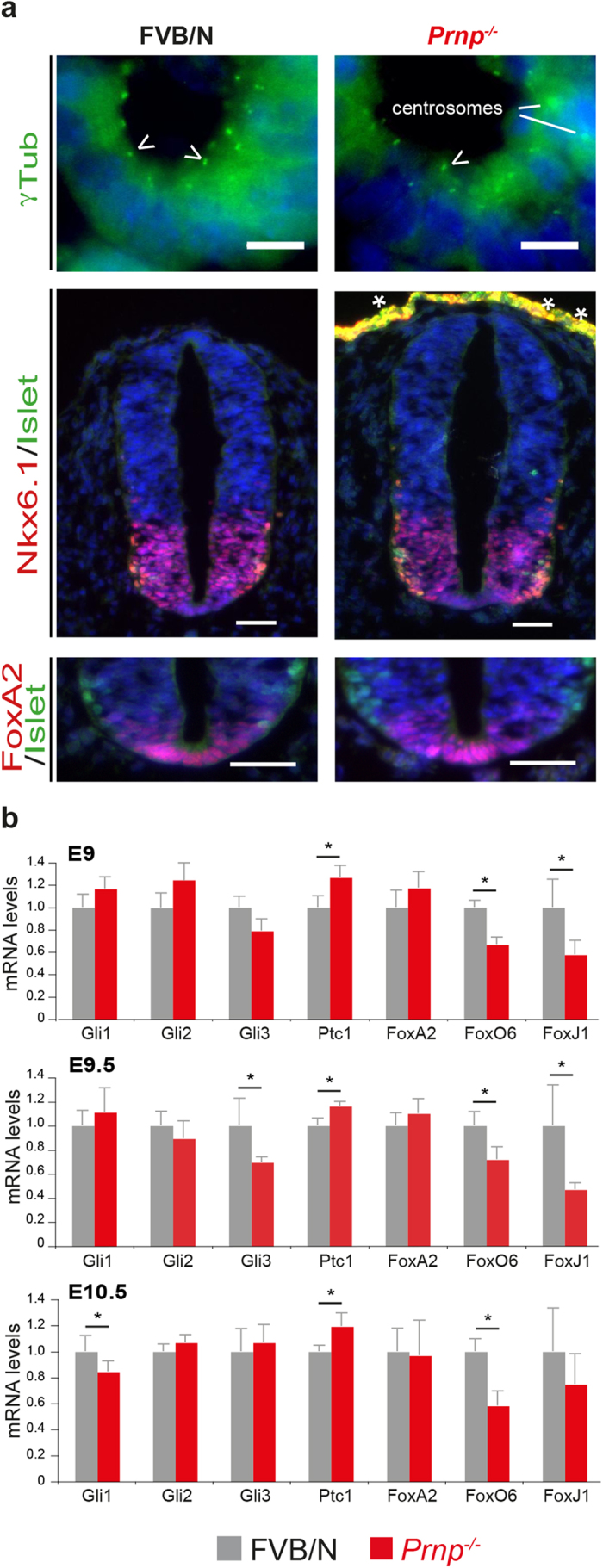
Dorsoventral patterning and Shh-related transcripts in the neural tube of PrP^C^-ablated embryos. Immunofluorescence analysis of transverse sections from FVB/N and *Prnp*^*−/−*^ E9 mouse embryos at the level of the mid-trunk region (**a**, representative images of n = 3 embryos analysed). Nuclei are stained with DAPI (blue). Arrowheads point to basal bodies. The asterisks indicate artefact signals. Scale bar: 50 μm, except for γTub staining, 10 μm. qPCR results showing the expression of Shh-related transcription factors and the Shh receptor Patched 1 (Ptc1) in FVB/N and *Prnp*^*−/−*^ neural tube-enriched preparations from E9 to E10.5 are shown (**b**, *n* ≥ *5*; *p < 0.05).

We next performed similar analyses in the embryo neuroepithelium, where primary cilia functions have been well documented^29^. Although no gross ultrastructural abnormalities (Fig. S4) or quantitative differences in the number of ciliary basal bodies were observed in the floor plate region between FVB/N and *Prnp*^*−/−*^ embryos at E9 or E9.5 (Fig. 4A; Table S1), PrP^C^ ablation exerted a recurrent impact on the expression of a number of Shh-related genes in neural tube-enriched preparations from E9 to E10.5 (Fig. 4b). Thus, Gli1 and Gli3 mRNA levels were slightly but significantly decreased by 20% and 30% at E10.5 and E9.5, respectively. In line with the *in vitro* data, Ptc1 was upregulated by 20%, and FoxO6 mRNA levels were significantly reduced by 30–40% throughout the time window of analysis. Finally, FoxJ1 expression, another forkhead family transcription factor upregulated by Shh signalling, was also decreased by up to 40–50% at E9 and E9.5.

Primary cilia and Shh signalling are critical for proper patterning of both the neural tube along the dorsoventral axis^28^ and the heart^36^. These were negligibly affected in *Prnp*^*−/−*^ E9 embryos, as shown by the conserved expression patterns of FoxA2 and Nkx6.1, another Shh pathway-dependent transcription factor (Fig. 4a and data not shown). Consistently, PrP^C^ ablation had no significant effects on FoxA2 mRNA levels over the E9–E10.5 period (Fig. 4b). The number of Islet-positive cells (an early Shh-dependent marker of differentiation) in the neural tube was also stable between the two genotypes at E9 (Fig. 4a, Table S1).

Regarding cell cycling, the mitotic indexes in the neural tubes of FVB/N and *Prnp*^*−/−*^ E9 embryos were similar (Table S1), and the cyclin D1 mRNA levels were conserved in FVB/N and *Prnp*^*−/−*^ neural tube-enriched preparations from E9 to E10.5 (Fig. S5).

Collectively, these data indicate that PrP^C^ depletion was detrimental to signalling functions related to primary cilium biology: in the 1C11 neuroepithelial cell line, the Shh signal transduction pathway was nearly switched off; in the developing mouse neural tube, the modest but consistent modulation of the transcript levels of effectors/regulators of the Shh pathway was accompanied by a robust decrease in the Shh targets FoxO6 and FoxJ1.

### PrP^C^ depletion affects the levels of α-tubulin variants

**Figure 5:**
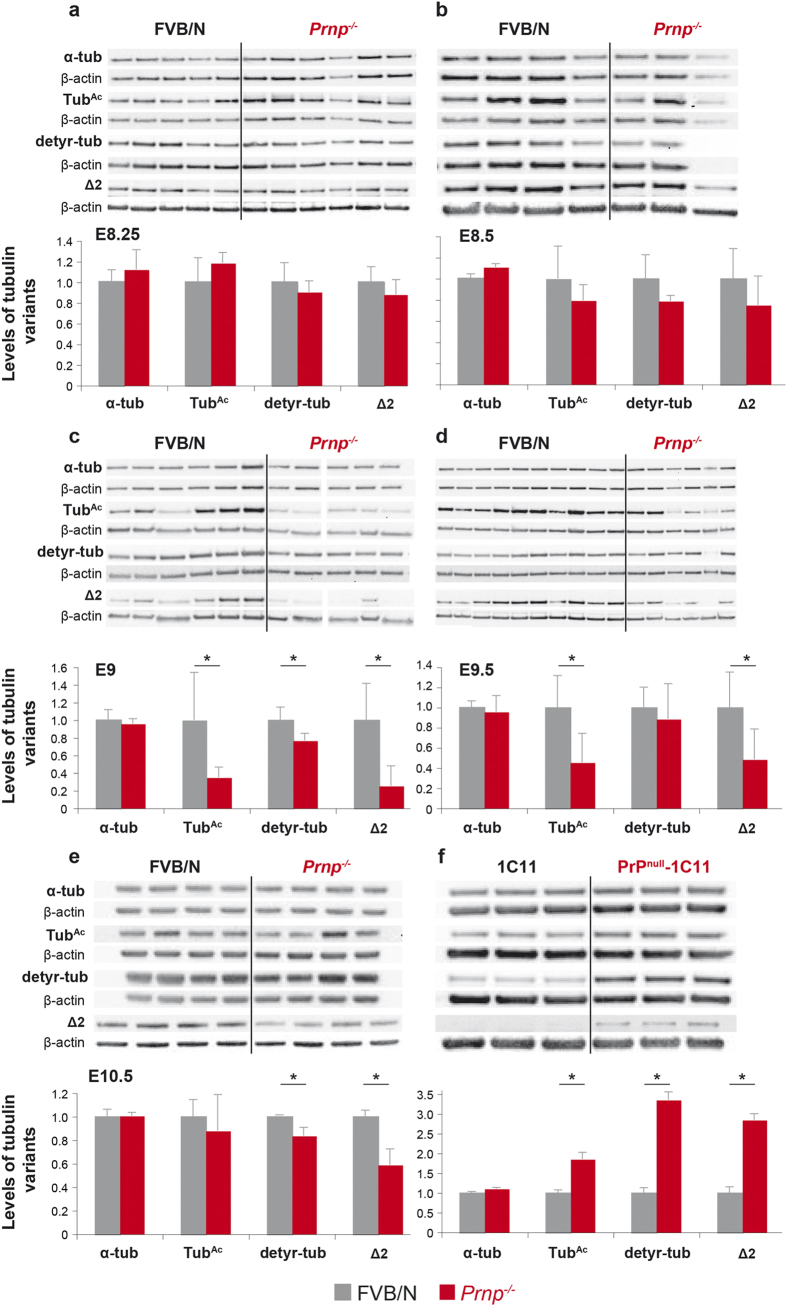
Analysis of α-tubulin post-translational modifications in developing embryos and 1C11 cells lacking PrP^C^. Immunoblot and densitometric quantification of total α-tubulin (α-tub), Tub^Ac^, detyr-tub and Δ2 variants in FVB/N embryos proper at E8.25 (**a**) and E8.5 (**b**), in neural tube-enriched preparations from embryos at E9 (**c**), E9.5 (**d**) and E10.5 (**e**) as well as in the 1C11 cell line (**f**) in the presence or absence of PrP^C^ (^*^p < 0.05). Immunoblot of protein extracts prepared from individual embryos or cell lysate are shown. The gels shown have been cropped for clarity and conciseness purposes, and have been run under the same experimental conditions; the original blots are shown in supplemental Figures S9 to S12.

The ciliary basal body is a microtubule organizing centre^37^ and the fine-tuning of cilium-dependent signalling pathways and regulation of microtubule dynamics are related processes^38^,^39^. Numerous subtypes of microtubules can be generated by tubulin PTMs, including acetylation, detyrosination, polyglutamylation and polyglycylation^40^. Tubulin PTMs involve a broad range of tightly regulated enzymes that are spatially and temporally key to microtubule assembly, stability and functions^40^ and thus emerge as key players in development. Detyrosinated α-tubulin (detyr-tub) is involved in proper neuronal organization^41^. The Δ2 variant, which is generated from detyr-tub by cytosolic carboxypeptidase cleavage^42^ (Fig. S6), is specifically enriched in neuronal cells and is linked to microtubule stability^43^. We thus assessed the impact of PrP^C^ depletion on the levels of total α-tubulin (α-tub), Tub^Ac^, detyr-tub, and Δ2 in the embryo proper (E8.25 and E8.5), in neural tube-enriched preparations (E9 to E10.5 embryos) and in 1C11 cells (Fig. 5). The numbers of litters and of embryos per litter analysed are indicated in the supplementary experimental procedures. Although the levels of α-tub did not exhibit significant variations, the levels of the Tub^Ac^, detyr-tub and Δ2 variants varied markedly between PrP^C^-expressing and PrP^C^-depleted groups both *in vivo* (from E9 onwards) and *in vitro*. *In vivo*, despite noticeable individual variations, there was a transient and significant decrease in the mean levels of Tub^Ac^ and Δ2 in *Prnp*^*−/−*^ samples at E9 and E9.5, compared with wild-type counterparts (Fig. 5c,d). At E9, the values were lowered by 3- to 4-fold. The levels of Δ2 in *Prnp*^*−/−*^ samples were still decreased at E10.5 (Fig. 5e). At E9 (Fig. 5c) and E10.5 (Fig. 5e), the levels of detyr-tub were also modestly but significantly decreased in *Prnp*^*−/−*^ versus FVB/N neural tube-enriched embryos. In 1C11 cells, the mean levels of the three analysed α-tubulin variants were also affected by PrP^C^ depletion (Fig. 5f). However, unlike the neural tube-enriched preparations, those levels were significantly increased by 2–3 fold.

Collectively, these data indicate that PrP^C^ depletion affects the steady-state levels of α-tubulin variants.

### Blockade of primary cilium resorption does not reproduce a PrP^null^-like phenotype

**Figure 6:**
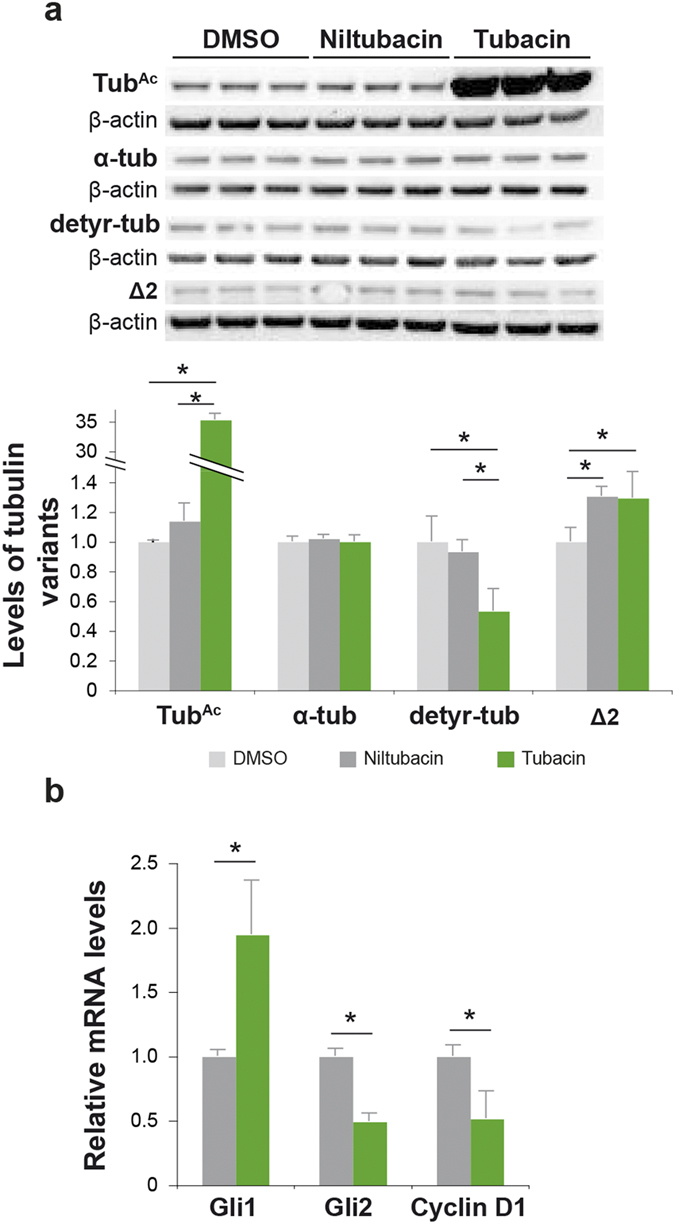
Hallmarks of primary cilium biology upon treatment of 1C11 cells with tubacin. 1C11 cells treated with tubacin, niltubacin and DMSO were compared with regards to protein levels of α-tub, Tub^Ac^, detyr-tub and Δ2 (**a**, *n* = *3*). qPCR results showing the expression of Gli transcription factors and Cyclin D1 are shown (**b**, *n* = *3*) (^*^p < 0.05). The gels shown have been cropped for clarity and conciseness purposes, and have been run under the same experimental conditions; the original blots are shown in supplemental Figure S8b.

Upon PrP^C^ depletion, the three α-tubulin variants were downregulated in neural tubes yet upregulated in 1C11 cells. The increased proportion of ciliated cells (Fig. 3a) in the PrP^null^-1C11 population (which may suggest a defect in primary cilium disassembly^22^) may contribute to the increase in tubulin variants^44^, to the diminished proliferation rate and possibly to alterations of the Shh pathway due to the coordinated/intertwined nature of these processes^28^,^45^. To investigate this possibility, we artificially maintained 1C11 primary cilia in a PrP-expressing context by treatment with tubacin. Tubacin is a selective inhibitor of histone deacetylase 6 (HDAC6)^46^,^47^, the predominant enzyme that deacetylates tubulin, a process necessary to deciliation^45^. As negative control, 1C11 cells were cultured in the presence of niltubacin, an inactive congener of tubacin. As previously reported^47^, tubacin treatment increased Tub^Ac^ levels by >30-fold compared to vehicle or niltubacin treatment (Fig. 6a). The detyr-tub and Δ2 variant levels were significantly decreased by ~2-fold and remained constant, respectively (Fig. 6a), an outcome contrasting with observations in PrP^null^-1C11 cells (Fig. 5f). We next used qPCR to compare the levels of Gli1, Gli2 and Cyclin D1 transcripts in 1C11 cells cultured in the presence of tubacin or niltubacin. Gli2 and Cyclin D1 expression was reduced by 2-fold, whereas Gli1 expression doubled in tubacin-treated cells (Fig. 6b). Thus, although tubacin affects Cyclin D1 expression similarly to PrP^C^ depletion, this treatment does not recapitulate the entire set of alterations of the Shh signalling pathway observed in PrP^null^-1C11 cells. These results suggest that PrP^C^ depletion, rather than impaired cilia resorption, is primarily driving the changes in tubulin PTM and Shh signalling observed in PrP^null^-1C11 cells. PrP^C^ depletion thus truly leads to different outcomes in cultured 1C11 cells and neural tubes as a whole with regard to α-tubulin PTM regulation.

### CCP1 and PrP^C^ co-localize in the embryo but the lack of PrP^C^ has no overt impact on CCP1 distribution

**Figure 7:**
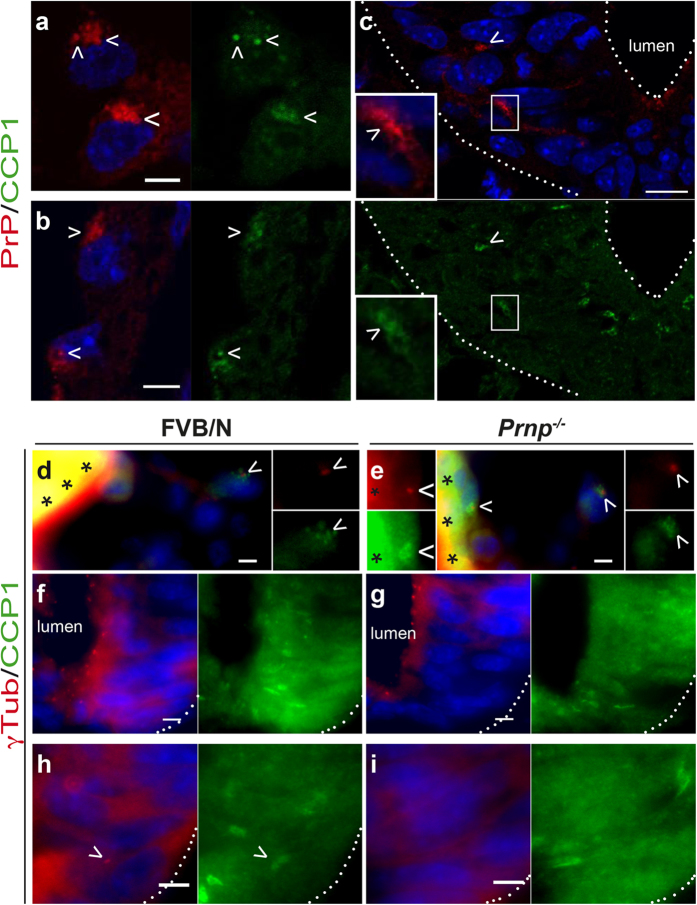
Detection of cytosolic carboxypeptidase 1 in the mouse embryo. Transverse sections from FVB/N (**a–d**,**f**,**h**) and *Prnp*^*−/−*^ (**e**,**g**,**i**) E9 mouse embryos co-stained for CCP1 (green), DAPI (blue) and PrP^C^ (**a–c**) or γTub (**d–i**) (red) are shown (representative images of n = 4 embryos analysed). The dotted lines and asterisks indicate the boundaries of the neural tube and artefact signals, respectively. PrP^C^ and CCP1 co-localization (arrowheads) in the yolk sac (**a**), omphalomesenteric artery (**b**) and the mantle zone of the neural tube (**c**, inset), as observed by confocal microscopy. Note that no co-localization is observed at the apical face of the floor plate (**c**). γTub and CCP1 co-staining in the yolk sac at the base of the primary cilium (arrowheads; **d**,**e**). Expression patterns of γTub and CCP1 in the neural tube (**f–i**). Juxtaposition is rarely observed (arrowheads in **h**). Scale bar: 5 μm, except in **c**: 10 μm.

Little is known about α-tubulin PTM regulation *in vivo*^40^,^48^. Acetylation primarily occurs on the Lys_40_ amino acid located on the microtubule lumenal surface, whereas tyrosination/detyrosination and subsequent removal of the penultimate glutamate residue to generate the Δ2 variant occur at the C-terminal tail of α-tubulin^40^,^42^,^48^ (Fig. S6). Although the enzymes responsible for the removal of the terminal tyrosine are unknown, cytosolic carboxypeptidases (CCPs), which are located in ciliary-based bodies (as PrP^C^) and other microtubule organizing centres^49^, are involved in the cleavage of detyr-tub to generate the Δ2 variant. This isotype was the most repressed isotype in *Prnp*^*−/−*^ neural tube-enriched preparations (Fig. 5). In addition, recombinant PrP has been reported to directly interact with the C-terminal domains of tubulin^50^. Collectively, these data led us to hypothesize that CCP might co-localize with PrP^C^. We thus probed mouse sections of FVB/N embryos at E9 (Fig. 7a–c) and E9.5 (data not shown) for PrP^C^ and CCP1, the only mouse CCP for which an antibody is available. In endothelial cells of the yolk sac (Fig. 7a) and the omphalomesenteric artery (Fig. 7b), CCP1 partially co-localized with PrP^C^ punctiform structures, and the signal juxtaposed with γ-tub labelling (Fig. 7d and data not shown), strongly suggesting that CCP1 localizes with PrP^C^ at the ciliary base.

In the neural tube, CCP1 co-localized with PrP^C^ in the mantle region but not at the ventricular face of the neuroepithelium (Fig. 7c). CCP1 and γ-tub labelling rarely overlapped (Fig. 7f,h), suggesting that in the neural tube, CCP1 localizes in zones of microtubule rearrangements distinct from the ciliary basal body. In the developing heart, no CCP1 labelling was observed (data not shown).

Our qPCR and immunoblot analyses of FVB/N versus *Prnp*^*−/−*^ neural tube-enriched preparations from E9.0 to E10.5 embryos (Fig. S7a–b) did not reveal significant variations in CCP1 levels, except for a modest reduction in CCP1 mRNAs at E10.5 (Fig. S7a). Finally, the distribution of CCP1 in the yolk sac (Fig. 7e) and in the neural tube (Fig. 7g,i) was not affected by PrP^C^ depletion at E9 and E9.5.

Taken together, these data suggest that although the lack of PrP^C^ does not affect CCP1 distribution, PrP^C^ and CCP1 co-localize at the ciliary base or in distinct zones of microtubule rearrangement, depending on the cell type and the differentiation state. These observations are consistent with a participatory role of PrP^C^ in the fine-tuning of Δ2-tubulin variant levels.

## Discussion

There is a fundamental and therapeutic interest in defining the physiological functions of the cellular form of the prion protein. In this work, we identify PrP^C^ as a ciliary base component in different types of stem cells and progenitors in the developing central nervous and cardiovascular systems. Using neuroepithelial cells displaying neuroectodermal progenitor hallmarks and mouse embryos, we further show that PrP^C^ depletion affects the fine-tuning of tubulin PTMs and cilium-related signalling.

Our data reveal that PrP^C^ is detected in the mouse embryo in neuroepithelial cells, in a stem cell niche associated with definitive haematopoiesis and in progenitors of the heart and blood vessels. In these structures, the protein is detected as soon as E8.25, i.e. around the same time period as *Prnp* expression^14^,^15^,^16^. These observations are consistent with reports linking PrP^C^ with the self-renewal of neural progenitors^10^ or haematopoietic stem cells^6^. We further document abundant PrP^C^ expression in the mantle zone of the developing neural tube, which is consistent with its involvement in neuronal differentiation^10^,^16^. Notably, the present PrP^C^ expression pattern is consistent with the biological pathways affected by *Prnp* invalidation, including cell adhesion, nervous system development, heart formation and angiogenesis^14^.

Although PrP^C^ has been briefly mentioned in two distinct ciliomes from choroid plexus epithelial cells^51^ and the outer segment of photoreceptor cells^52^, our imaging of E9–E9.5 mouse embryos unambiguously links PrP^C^ to the base of the primary cilium in the aforementioned stem cells and progenitors. PrP^C^ was not detected along the ciliary axoneme, excluding a general distribution at the ciliary membrane. Importantly, PrP^C^ does not localize to centrosomes in general but only at the ciliary base of a limited set of cells. In initial attempts to further characterize its subcellular distribution, PrP^C^ was found to co-localize with the recycling endosome marker Rab11, suggesting enrichment in the pericentriolar recycling endocytic compartment. Although a major proportion of PrP^C^ is detected as a lipid raft-clustered, cell surface protein in many differentiated cell types, this newly discovered intracellular topological localization is not unexpected. Indeed, a substantial fraction of PrP^C^ is known to cycle constitutively between the plasma membrane and endocytic compartments^2^. Whether PrP^C^ is localized in the ciliary pocket and/or in the membrane transition zone remains to be determined.

We propose that at the ciliary base, PrP^C^ contributes to ciliary function. Proper ciliary function is critical for neural stem cells located at the apical face of the neuroepithelium to sense signals such as Shh^29^. The participation of PrP^C^ in ciliary function is notably supported by the reduced levels of FoxJ1 in *Prnp*^*−/−*^ neural tubes, a Shh-induced transcription factor reported to regulate ciliogenesis^53^. Reduced FoxJ1 expression may reflect defects in Shh signalling, a hypothesis supported by the decreased levels of FoxO6, another Shh target gene.

The functional link between PrP^C^, cilia and Shh signalling was further corroborated by our data obtained for 1C11 neuroepithelial cells. In the 1C11-derived PrP null cells, PrP^C^ has been knocked down by an RNAi approach, thus ruling out any contribution of *Prnp*-flanking genes to the resultant phenotype^54^. The PrP^C^ knockdown drastically affected cilium turnover and cell cycle regulation, as well as Shh signalling. The dysregulation of these intertwined processes was not merely a by-product of primary cilium impaired resorption, as shown by tubacin treatment. The more limited effect of PrP^C^ depletion on ciliary functions *in vivo* is not unexpected, as overt impairment of primary cilium functions is usually associated with pleiotropic developmental abnormalities^22^,^29^, which are not observed in adult mice with inactive *Prnp*^11^,^12^. Notably, the alterations found in the Shh regulatory circuitry *in vivo* appear to affect genes that are important for differentiation (e.g., FoxJ1^55^) and subsequent synaptogenesis (e.g., FoxO6^56^) rather than for dorsoventral patterning of the neural tube (e.g., FoxA2^28^), which is consistent with mild but significant alterations. Indeed, FoxJ1 is important for floor plate cilia architecture, whereas its absence does not affect floor plate identity or ciliogenesis^57^. Furthermore, although viable and outwardly normal^56^, FoxO6 knockout mice exhibit impaired memory consolidation^56^, which recalls the phenotype of PrP knockout mice^58^.

A second important contribution of this study is the identification of a link between PrP^C^ and the fine-tuning of microtubule PTMs. While it is not yet possible to conclude on the direct involvement of PrP^C^ in microtubule PTM regulation, such a function would be truly consistent with PrP^C^ topological location, the primary cilium basal body being a microtubule organizing centre^37^. Thus, the levels of Tub^Ac^, detyr-tub and Δ2 α-tubulin isoforms were markedly decreased in *Prnp*^*−/−*^ neural tube-enriched preparations, at a stage in which PrP^C^ begins to be broadly detected in the developing CNS. PrP^C^ depletion in 1C11 cells yielded prominent, albeit opposite, changes in tubulin PTMs. Those changes were not driven by the concomitant defects in primary cilia disassembly. The limited time window for the PTM response to PrP^C^ depletion *in vivo* would lend support for dynamic mechanisms of adaptation, which may not occur or may occur differently in 1C11 cells, as recently documented in Shh signalling^59^. Moreover, the components of the pathway or the PrP^C^ interacting partners may qualitatively differ between the neural tube and 1C11 cells, resulting in opposite effects, as previously observed with respect to neurite outgrowth^60^,^61^. These mechanisms of regulation or adaptation may also vary according to the cellular differentiation state. In this respect, it is worth mentioning that data obtained using neural-tube enriched preparations cannot discriminate between neural progenitors and differentiating neurons, in contrast with 1C11 cells.

The tubulin variants studied here appear to be important in both cell contexts. They may, on the one hand, participate in ciliary maintenance in both progenitors and differentiating cells. On the other hand, their accumulation appears to accompany neuronal differentiation^40^. Their deregulation in PrP^C^-deficient progenitors may thus affect ciliary functions, as suggested in PrP^null^-1C11 cells and also by the alterations in Shh downstream targets *in vivo*. Because certain tubulin PTM alterations persist at later stages (E10.5) in the neural tubes of *Prnp*^*−/−*^ embryos, those alterations may also affect neuronal differentiation, in accordance with the contribution of PrP^C^ to this process. In direct support of this hypothesis, we found drastically reduced expression of the neuronal differentiation marker NFL in the neural tubes of E10.5 *Prnp*^*−/−*^ embryos (SML, unpublished observations). Refining the link between PrP^C^ and tubulin PTM may thus yield further insight into how PrP^C^ contributes to ciliary function and neuronal differentiation.

How PrP^C^ might regulate α-tubulin PTM mechanistically in the developing mouse embryo remains an open question that is further obscured by the absence of a coherent picture of the generation and enzymatic regulations of these PTMs and by the dynamic nature of their interactions^40^,^48^. We conducted a series of preliminary experiments on the expression pattern of CCP1 (which had not been previously documented in the mouse embryo), a recently described cytosolic carboxypeptidase responsible for the generation of Δ2-tubulin, the mostly altered α-tubulin isoform in neural tube-enriched preparations from PrP-null mice. CCP1 was found to co-localize with PrP^C^ in yolk sac vessels, the omphalomesenteric artery and the mantle zone of the neural tube. PrP^C^ could thus participate in CCP1 activity through a signalling cascade/platform, as commonly observed with this protein^3^,^4^,^5^. Alternatively, as recombinant PrP can interact directly with the C-terminal tail of tubulin^50^, where Δ2 generation occurs^40^,^48^, PrP^C^ may directly regulate CCP1 access to the C-terminal domains of tubulin. Such regulation (i.e., CCP1 enzymatic activity) would be consistent with the absence of any significant effect of PrP^C^ ablation on the subcellular localization or the protein levels of CCP1. *In vitro* studies with purified components will help to determine whether these interactions do physically occur.

In summary, our study links PrP^C^ to the primary cilium biology and to the developmental regulation of microtubule subtypes, two tightly coordinated processes controlling stem cell fate. This information will certainly help to elucidate how PrP^C^ and differentiation intersect mechanistically. The emerging links between microtubule PTM regulation and neurodegeneration^42^ and evidence that a significant number of signalling pathways altered in *Prnp*^*−/−*^ embryos are over-activated at late stage of prion disease pathogenesis^14^ warrant the study of possible alterations in microtubule dynamics during PrP^C^-dependent neurodegeneration in prion diseases and Alzheimer’s disease.

## Methods

### Mouse

Animal experiments were conducted in strict accordance with EU directive 2010/63 and were approved by the local ethics committee of the authors’ institution (Comethea; permit number 12/034). FVB/N *Prnp*^*−/−*^ mice were kindly provided by S.B. Prusiner (UCSF, San Francisco). E8.25 to E10.5 FVB/N and FVB/N *Prnp*^*−/−*^ embryos were dissected in ice-cold phosphate buffered saline (PBS) and immediately frozen in liquid nitrogen for RNA and protein analysis or fixed for electron microscopy and immunofluorescence analyses.

### Cell culture, cell viability and cell cycle analyses

The 1C11 cells and their PrP^−null^ counterparts were grown as described previously^27^. Cell cycle, MTT assays and immunofluorescence analyses were performed as described in the supplemental procedures.

### Immunofluorescence microscopy

Mouse embryos were prepared and transverse sections were analysed by immunofluorescence as previously described^14^. Specific primary antibodies are described in Supplementary Table S2. Sections were imaged using an inverted microscope with epifluorescence illumination (Zeiss Axio Observer.Z1, France) and a cooled CDD camera (CoolSNAP HQ2; Photometrics, Roper Scientific, Lisses, France). Images were captured using AxioVision (Zeiss-France). Z-series were acquired on a Zeiss LSM 700 confocal microscope. Images were analysed with ImageJ and when necessary with the Bio-Formats plugin.

### Real-Time PCR

Total RNA from embryos or 1C11 cells was isolated using Trizol (Invitrogen, Cergy Pontoise, France) according to the manufacturer’s instructions. The corresponding cDNA was synthesized with oligo(dT)_17_ primer using 200 units of Superscript III™ reverse transcriptase (Invitrogen) according to the manufacturer’s protocol. Real-time PCR was performed using Absolute QPCR SYBR Green ROX Mix (Thermo-Scientific) on an ABI PRISM 7900HT (Applied Biosystems, Illkirsch, France). The sequences of the RT-PCR primers are given in Supplementary Table S3.

### Immunoblotting

Embryos proper, neural tube-enriched samples and 1C11 cells were homogenized in anti-protease (Sigma)-containing buffer (50 mM Tris-HCl, pH 7.4, 5 mM EDTA, 300 mM NaCl). The total protein concentration was determined by a bicinchoninic acid assay (Thermo-Scientific). 5–10 μg protein samples were then run on 12% Bis-Tris polyacrylamide gels (Bio-Rad, Marnes-la-Coquette, France), electrotransferred and blotted onto nitrocellulose membranes with the antibodies of interest (Table S2). Immunoreactivity was visualized by enhanced chemiluminescence (Amersham Pharmacia Biosciences, GE Healthcare Europe, Velizy-Villacoublay, France). The protein levels were quantified with the GeneTools software, after acquisition of chemiluminescent signals with a GeneGnome digital imager (Syngene, Frederick, Maryland, United States). The blots were also incubated with anti-β-actin antibody (Table S2) to normalize signals to β-actin as a loading control for quantification.

### Statistical analysis

Data are presented as the means of replicates (as indicated) ± SDs. Significant differences between groups were examined using the nonparametric Mann-Whitney and Wilcoxon tests (small sample tests; Analyse-it software) unless specified otherwise, and *p*-values <0.05 were considered significant.

## Additional Information

**How to cite this article**: Halliez, S. *et al.* Prion protein localizes at the ciliary base during neural and cardiovascular development, and its depletion affects α-tubulin post-translational modifications. *Sci. Rep.* **5**, 17146; doi: 10.1038/srep17146 (2015).

## Supplementary Material

Supplementary Information
