# Supplementary material for: Prion protein localizes at the ciliary base during neural and cardiovascular development, and its depletion affects α-tubulin post-translational modifications
Source: Sci Rep. 2015 Dec 18;5:17146. doi: 10.1038/srep17146 (PMC4683536; doi:10.1038/srep17146)
Supplement: Supplementary Information [file srep17146-s1.doc]

**Supplementary Information**

**Prion protein localizes at the ciliary base during neural and cardiovascular development, and its depletion affects α-tubulin post-translational modifications**

Sophie Halliez^1*^, Séverine Martin-Lannerée^2^, Bruno Passet^3^, Julia Hernandez-Rapp^2^, Johan Castille^3^, Céline Urien^1^, Sophie Chat^3,4^, Hubert Laude^1^, Jean-Luc Vilotte^3^, Sophie Mouillet-Richard^2^ and Vincent Béringue^1*^

^1^INRA (Institut National de la Recherche Agronomique), UR892, Virologie Immunologie Moléculaires, Jouy-en-Josas, France

^2^INSERM UMR-S1124, Université Paris Descartes 75006, Paris, France

^3^INRA, UMR1313, Génétique Animale et Biologie Intégrative, Jouy-en-Josas, France

^4^INRA, Plateforme MIMA2, Jouy-en-Josas, France

*Corresponding authors: vincent.beringue@jouy.inra.fr; sophie.halliez@jouy.inra.fr

**Inventory of Supplementary Information**

Figure S1

Figure S2

Figure S3

Figure S4

Figure S5

Figure S6

Figure S7

Figure S8

Figure S9

Figure S10

Figure S11

Figure S12

Table S1

Table S2

Table S3

Supplemental Experimental Procedures

##
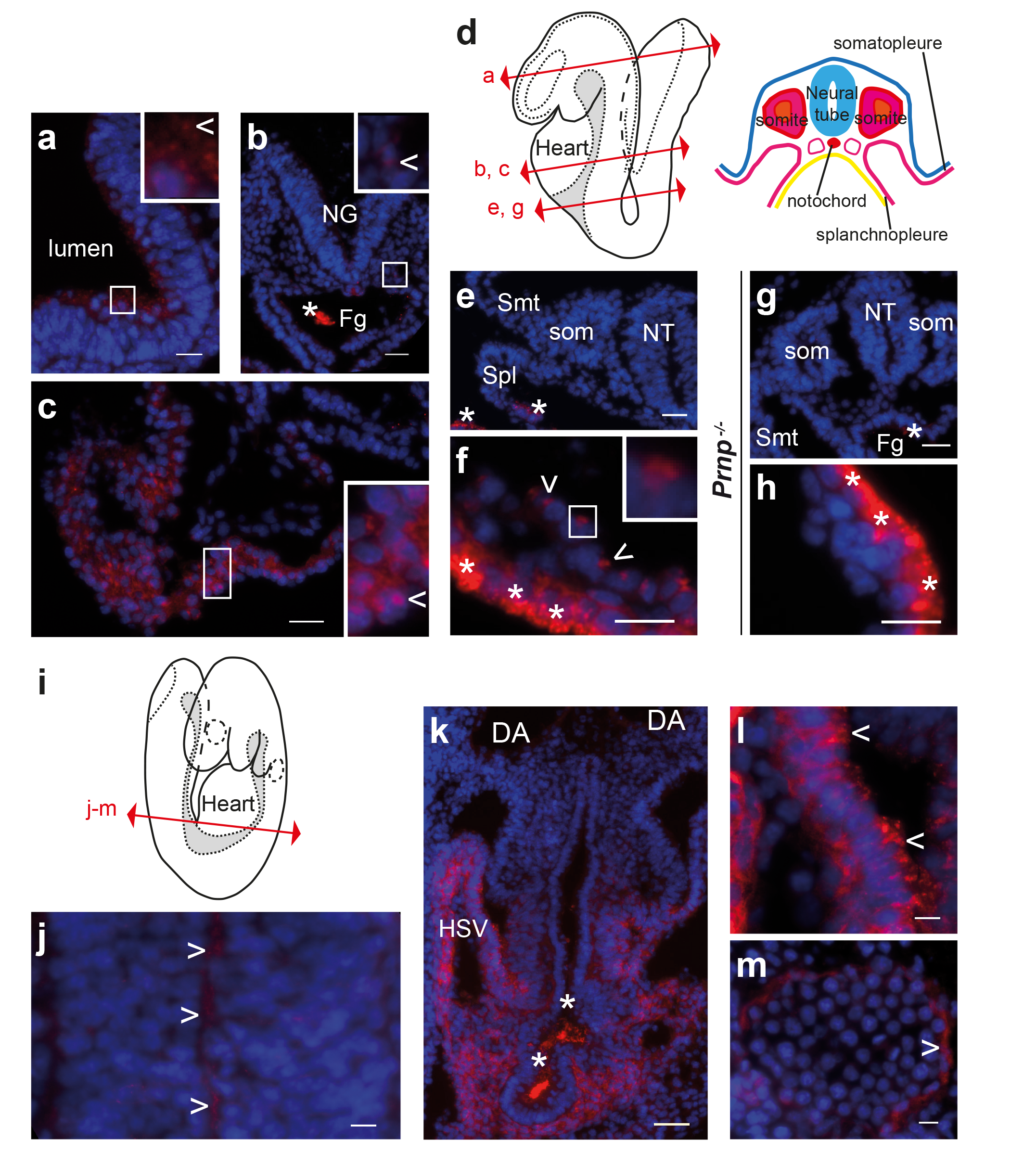


**Figure S1. PrP^C^ expression pattern in early developing mouse embryos.** PrP^C^ (red) and nuclear marker 4’,6-diamidino-2-phenylindole (DAPI, blue) staining of transverse sections from FVB/N (**a–c**, **e–f**, **j–m**) and FVB/N-*Prnp^-/-^* (**g–h**) mouse embryos at E8.25 (**a–c**, **e–h**) and E9 (**j–m**). Section plans are indicated for E8.25 (**d**) and E9 (**i**). Throughout the panel of images shown, the arrowheads indicate PrP-positive patches and punctuate structures, and the asterisks indicate artefact signals found in FVB/N as in *Prnp*^-/-^ mice (e.g. **g–h**), and likely due to non specific Fc binding to maternal blood. Immunofluorescence of E8.25 embryos at the level of the optic evagination (**a**), the neural groove (NG) (**b**), the embryonic heart ventricle (**c**), the mid-trunk region showing embryo proper and extra-embryonic regions (**e, g**) and details of yolk sac (**f, h**). Immunofluorescence of the developing nervous system (detail of the neural tube; **j**) and the developing cardiovascular system from E9 embryos (**k–m**) are shown. Staining of the venous pole of the heart at low (**k**) and high magnification (**l**). Image showing faint PrP^C^ expression in the endothelium of the dorsal aorta (DA) (**m**).

Scale bar: 10 µm (**j, l, m**), 20 µm (**a, f, h**), 30 µm (**b, c, e, g**), 50 µm (**k**).

Fg: foregut, HSV: horn sinus venosus, NT: neural tube, Smt: somatopleure, som: somite, Spl: splanchnopleure.

**
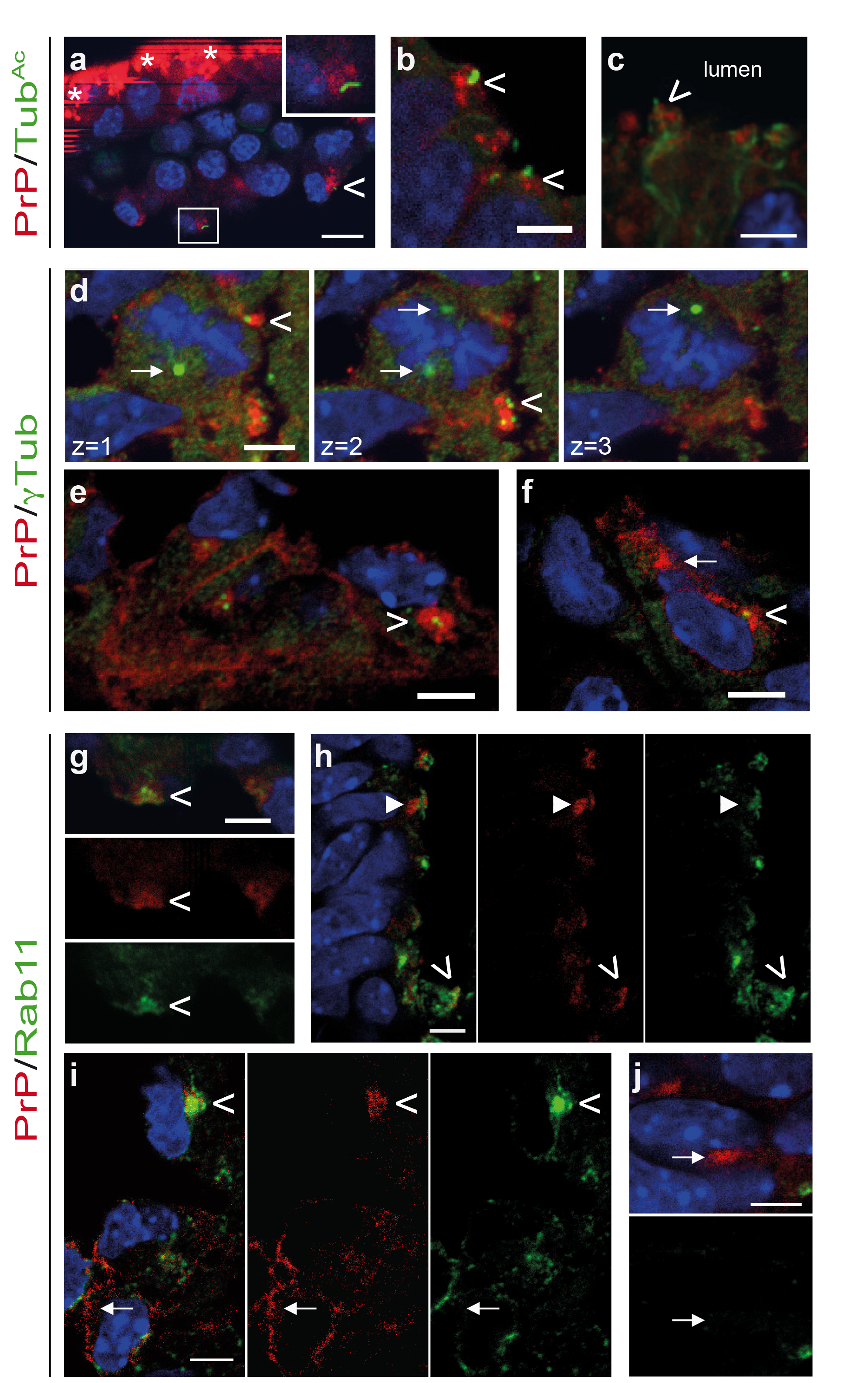
**

**Figure S2. PrP^C^ localization at the base of the primary cilium in stem and progenitor cells from the developing nervous and cardiovascular systems.** Confocal microscopy imaging of transverse sections of FVB/N mouse embryos at E9-9.5 co-stained for PrP (red) and (green) acetylated tubulin (Tub^Ac^) (**a–c**), γ-tubulin (γTub) (**d–f**) and Rab11 (**g–j**). Nuclei are stained with DAPI (blue). Merged confocal images or individual channels are shown. Z-series (**d**) or single optical sections (**a-c**, **e-j**) are presented. Detection of PrP^C^ at the base of Tub^Ac^–positive primary cilia (arrowheads) in endothelial cells in a yolk sac capillary (**a**) and at the venous pole (**b**). The asterisks indicate artefact signals. At the apical face of the neuroepithelium (forebrain), PrP^C^ is often found at the vicinity of Tub^Ac^-positive structures corresponding morphologically to primary cilia (**c**, arrowhead). PrP^C^ co-localizes with γTub corresponding to ciliary bases at the venous pole (**d**, arrowheads). White arrows point to the centrosomes of a mitotic cell and highlight the absence of detectable PrP^C^ signal.

Co-localization of PrP^C^ with γTub (**e**, arrowhead) in cells from the myocardium**.** In the mantle zone of the neural tube (**f**), PrP^C^ co-localized with γTub (arrowhead) but not systematically (white arrow). Co-localization (arrowheads) or co-regionalization (plain arrowheads) between PrP^C^ and Rab11 at the capillary of the yolk sac (**g**), at the venous pole (**h**) and in the myocardium (**i**). The white arrows point to areas where PrP^C^ and Rab11 do not co-localize in the mantle zone of the neural tube (**j**) or upon PrP^C^ expression at the cell surface in the myocardium (**i**).

Scale bar: 5 µm; except in **A**: 10 µm.

**
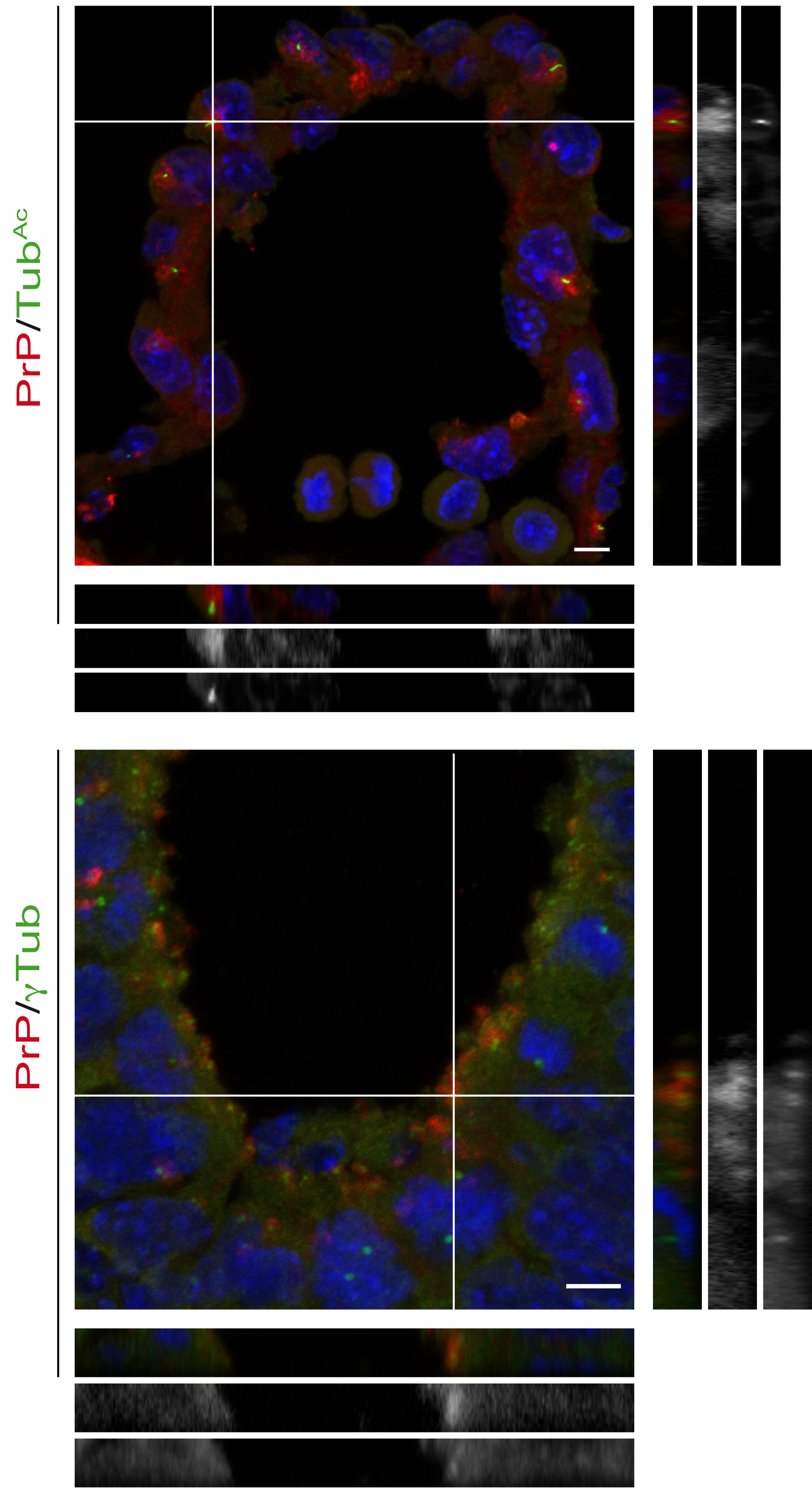
**

**Figure S3. PrP^C^ localization at the base of the primary cilium in the omphalomesenteric artery and at the floor plate.** Confocal microscopy imaging of the omphalomesenteric artery (top) and the floor plate of the neural tube (hindbrain; bottom) from FVB/N mouse embryos at E9-9.5 co-stained for PrP (red) and (green) acetylated tubulin (Tub^Ac^) (top) or γ-tubulin (γTub) (bottom). Nuclei are stained with DAPI (blue). Merged stack projections along the Z-axis and orthogonal views (XZ and YZ plans) are shown.

**
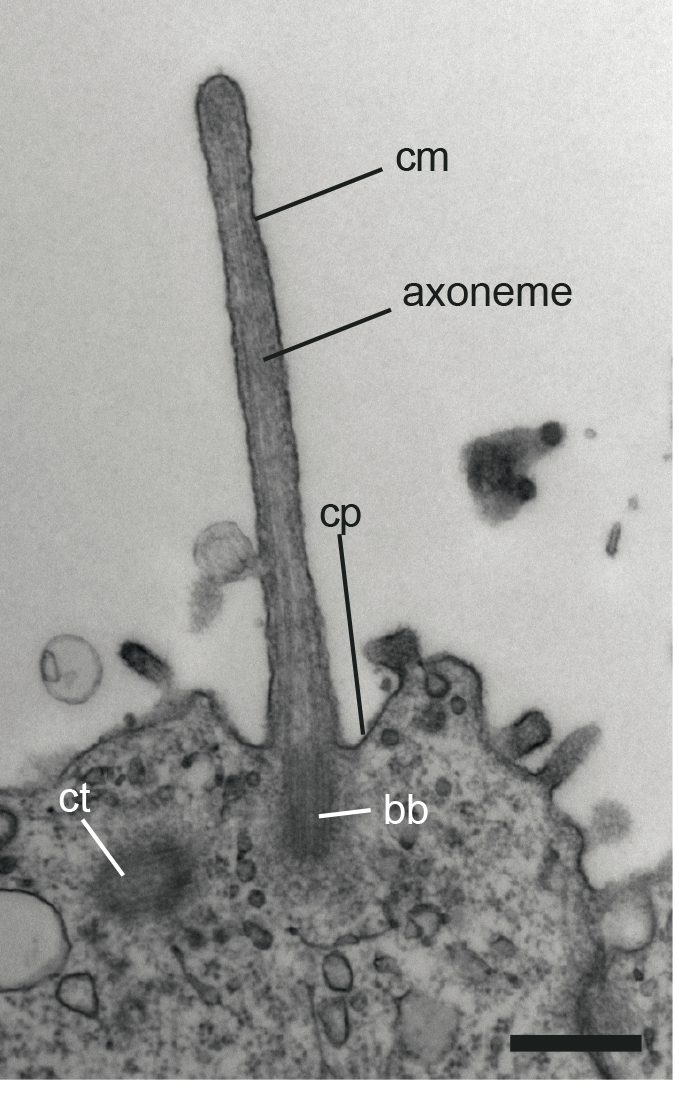
**

**Figure S4. Electron microscopy of a primary cilium in the floor plate region from E9.5 *Prnp^-/-^* mouse embryos.**

cm.: ciliary membrane, cp: ciliary pocket, bb: basal body, ct: centriole

Scale bar: 500 nm.


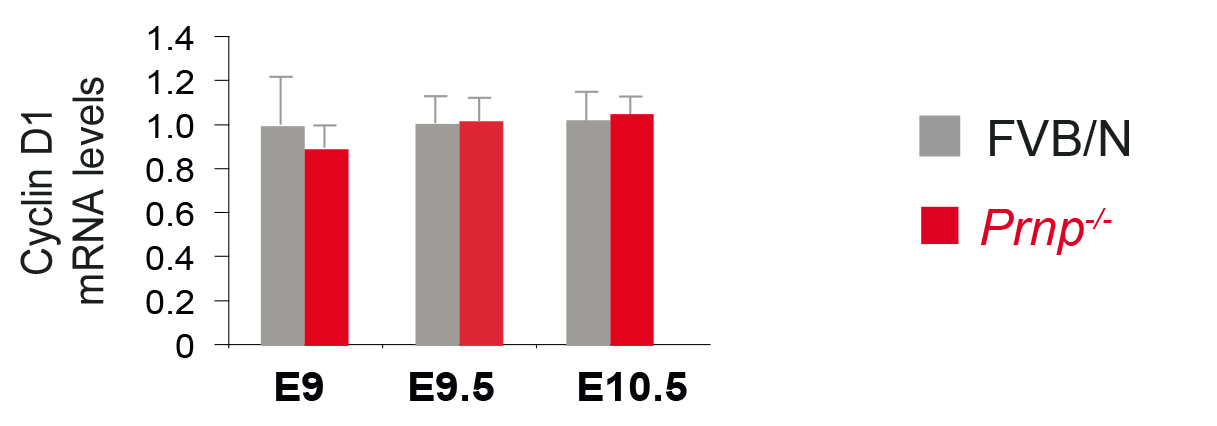


**Figure S5. Cyclin D1 mRNA levels in the neural tube of mouse embryos expressing or not PrP^C^.** qPCR results showing the expression of Cyclin D1 in FVB/N and *Prnp^-/-^* neural tube-enriched preparations from E9 to E10.5 are presented (*n≥5*).


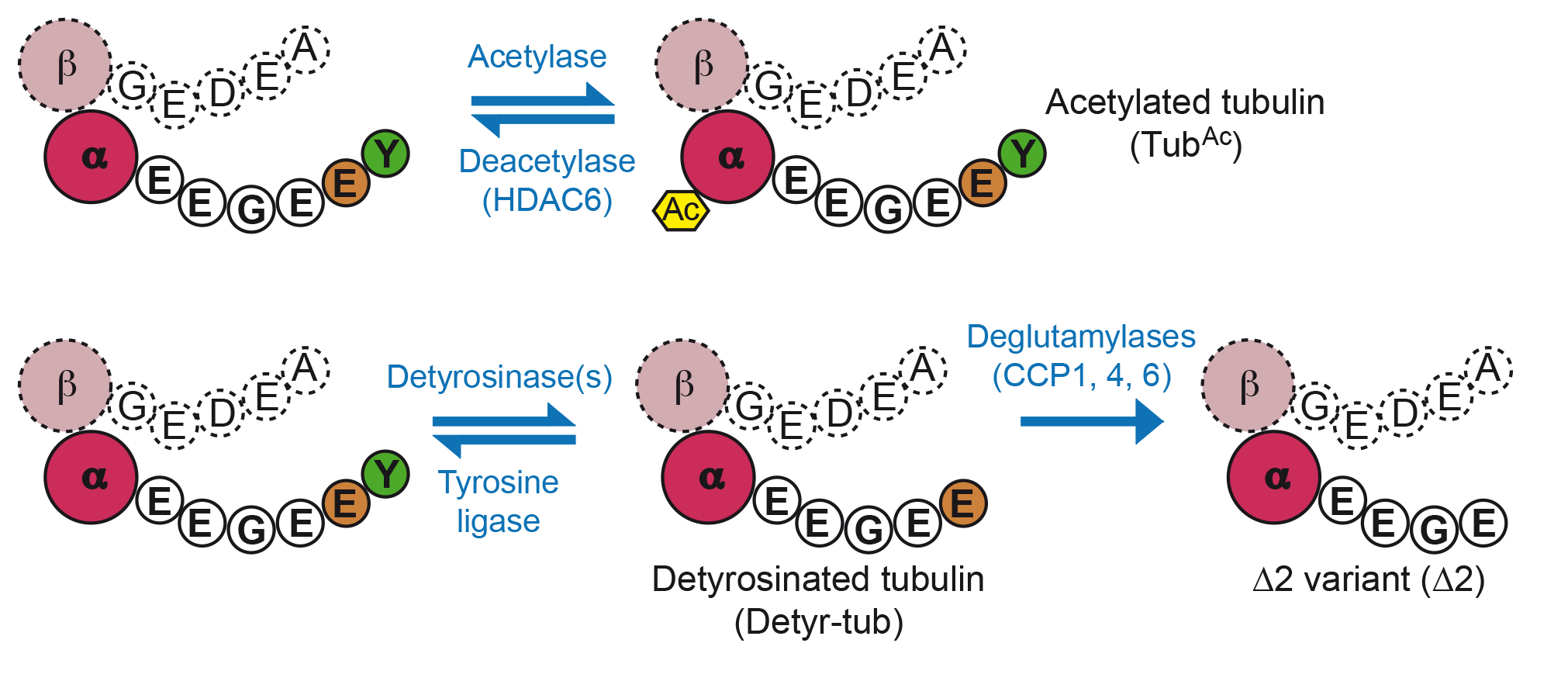


**Figure S6. Selective α-tubulin post-translational modifications (PTMs).** Schematic representation of the α-tubulin PTMs relevant to this study and of the enzymes involved. Note that the enzyme responsible(s) for the detyrosination of the α-tubulin has (/have) not been identified yet, and that the generation of the Δ2 variant is irreversible.


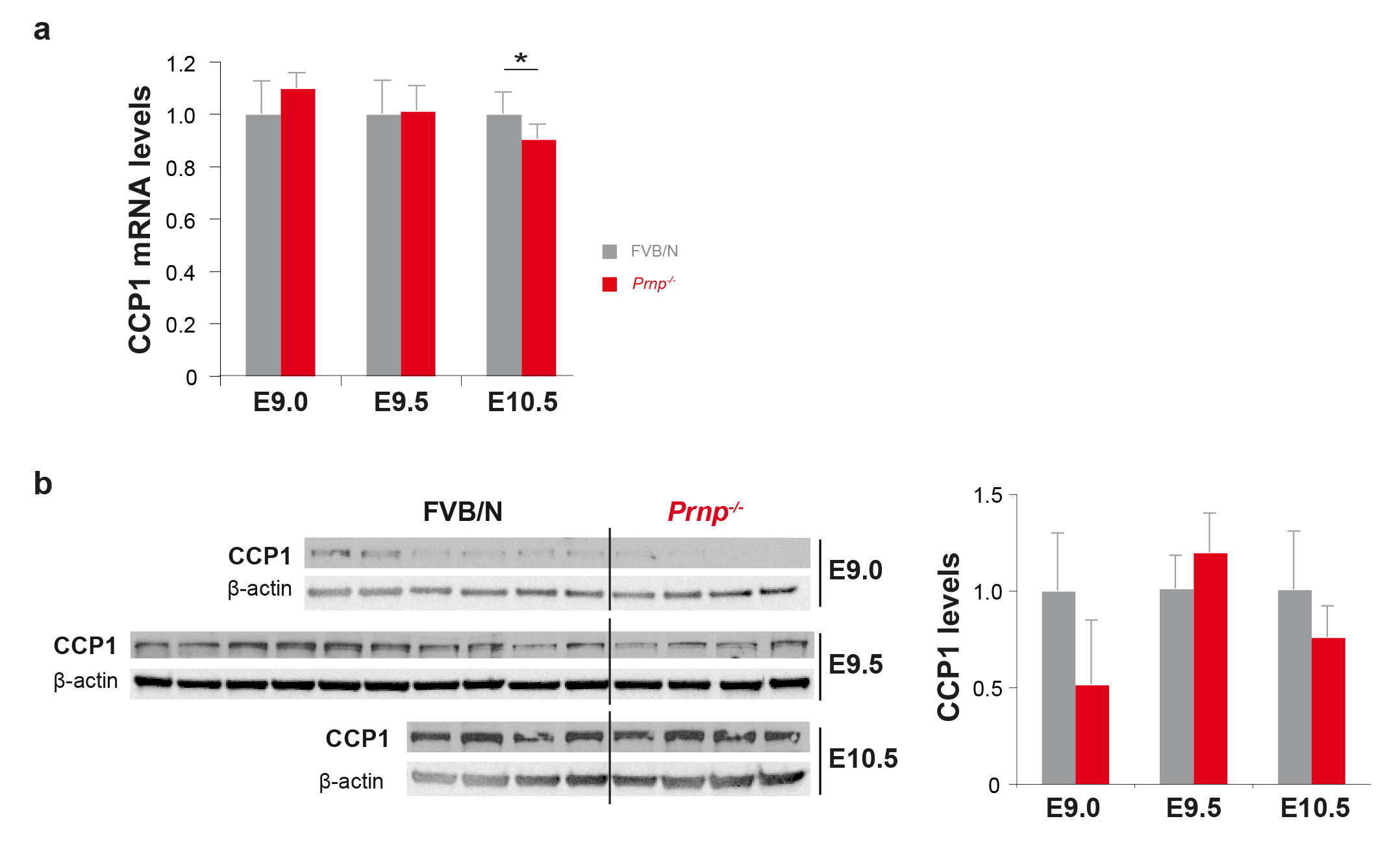


**Figure S7. CCP1 expression in early developing mouse embryos expressing or not PrP^C^.**

qPCR results (**a**) and immunoblot analyses (**b**) showing CCP1 expression in neural tube-enriched preparations from E9 to E10.5 embryos expressing or not PrP^C^ are presented (*n≥5*). (*: *p*<0.05).

**Figure S8. Uncropped gels from figure 3 and figure 6.**

1. uncropped gels from figure 3.
2. Uncropped gels from figure 6. The exposure time is indicated.

**Figure S9. Uncropped gels from figure 5.**

An X indicates that the sample was not incorporated the figure. The exposure time is indicated.

**Figure S10. Uncropped gels from figure 5.**

An X indicates that the sample was not incorporated the figure. The exposure time is indicated.

**Figure S11. Uncropped gels from figure 5.**

An X indicates that the sample was not incorporated the figure. The exposure time is indicated**.**

**Figure S9. Uncropped gels from figure 5.**

An X indicates that the sample was not incorporated the figure. The exposure time is indicated.

**Table S1: Wilcoxon signed ranks tests of difference between FVB/N and *Prnp^-/-^* embryos**

| **Variables** | **Signed ranks** | ***N*** | **Mean rank** | **Sum of ranks** | ***Z* value** | **Significance level** |
| --- | --- | --- | --- | --- | --- | --- |
| **Number of basal bodies in floor plate region at E9** | Negative  Positive  Ties  Total | 8  5  3  16 | 7,4  6,3 | 59,5  31,5 | -0,978 | n.s. |
| **Number of basal bodies in floor plate region at E9.5** | Negative  Positive  Ties  Total | 11  11  0  22 | 11,2  11,8 | 123  130 | 0,114 | n.s. |
| **Mitotic index in floor plate region at E9** | Negative  Positive  Ties  Total | 5  5  0  10 | 5  6 | 25  30 | 0,255 | n.s. |
| **Mitotic index in neural tube at E9** | Negative  Positive  Ties  Total | 5  5  0  10 | 7,6  3,4 | 38  17 | -1,07 | n.s. |
| **Number of Islet-positive cells in neural tube at E9** | Negative  Positive  Ties  Total | 13  9  0  22 | 12,7  9,7 | 165,5  87,5 | -1,266 | n.s. |

n.s.: not significant

The number of Islet-positive cells in neural tubes were quantified on transverse sections of E9 FVB/N and *Prnp^-/-^* embryos, after labelling with anti-Islet antibody (3 embryos per genotype). FVB/N and *Prnp^-/-^* embryo sections were paired according to the level of the section along the antero-posterior axis.

**Table S2: list of antibodies used**

| **IMMUNOFLUORESCENCE** | | |
| --- | --- | --- |
| **Sha31** | Mouse IgG1 | 1 |
| **Anti-acetylated tubulin** | Mouse IgG2b | Sigma |
| **Anti-γ-tubulin** | Mouse IgG1 | Sigma |
| **Anti-γ-tubulin** | Rabbit | Abcam |
| **Anti-Rab11** | Mouse IgG2a | BD |
| **Anti-phospho-Histone H3** | Rabbit | Upstate |
| **Anti-CCP1** | Rabbit | Proteintech |
| **Anti-FoxA2 (**4C7) | Mouse IgG1 | Developmental Studies Hybridoma Bank |
| **Anti-Nkx6.1 (**F55A10) | Mouse IgG1 | Developmental Studies Hybridoma Bank |
| **Anti-Islet-1/2 (**39.4D5) | Mouse IgG2b | Developmental Studies Hybridoma Bank |
| **IMMUNOBLOTTING** | | |
| **Anti-Cyclin D1** | Rabbit | Cell Signaling Technology |
| **Anti-α-tubulin** | Mouse IgG1 | Sigma |
| **Anti-α-tubulin** | Mouse IgG1 | Millipore |
| **Anti- acetylated tubulin** | Mouse IgG2b | Sigma |
| **Anti-detyrosinated tubulin** | Rabbit | Chemicon International |
| **Anti-∆2 tubulin** | Rabbit | Millipore |
| **Anti-β-actin** | Mouse IgG2a | Sigma |
| **Anti-CCP1** | Rabbit | Proteintech |

**Table S3: Primer sequences used for qPCR**

| **Gli1** | forward | 5’-TCCTAAAGAAGGGCTCATGGTA-3’ |
| --- | --- | --- |
|  | reverse | 5’-TCGACCTGCAAACCGTAATCC-3’ |
| **Gli2** | forward | 5’-TTTGCCGATTGACATGAGACA-3’ |
|  | reverse | 5’-GGTGGGAGGCCCGTGTAC-3’ |
| **Gli3** | forward | 5’-AACCCTATTCTACCCTCCAAA-3’ |
|  | reverse | 5’-GCTGATAGTGCTGGTATTGCT-3’ |
| **Ptc1** | forward | 5’-CTTCTGGGAAGGGGCAAA-3’ |
|  | reverse | 5’-ATCTGGGTCGGCTGGGTT-3’ |
| **Smo** | forward | 5’-AGACTCCGTGAGTGGCATCTGTTT-3’ |
|  | reverse | 5’-AGGGTGGTTGCTCTTGATGGAGAA-3’ |
| **FoxA2** | forward | 5’-ACACGCCAAACCTCCCTAC-3’ |
|  | reverse | 5’-GGGCACCTTGAGAAAGCA-3’ |
| **FoxO6** | forward | 5’-GGGCTGAGTCGAAGTTGAAG-3’ |
|  | reverse | 5’-GACGTCGAGTCCATCATCCT-3’ |
| **FoxJ1** | forward | 5’-AGCAAGGCCACCAAGATCACT-3’ |
|  | reverse | 5’-CGAGGCACTTTGATGAAGCA-3’ |
| **CycD1** | forward | 5’-CAGAAGTGCGAAGAGGAGGTC-3’ |
|  | reverse | 5’-TACTCTTAGAGGCCACGAACAT-3’ |
| **CCP1** | forward | 5’-AAAACCGCCTTCCGCTCCCC-3’ |
|  | reverse | 5’-CATCGTCCACTTCAGGCGGCA-3’ |

## SUPLEMENTAL EXPERIMENTAL PROCEDURES

### Cell culture reagents

All tissue culture reagents were from Invitrogen (Carlsbad, CA, USA). Tubacin and niltubacin were from Selleck Biochemical (Houston, TX, USA).

### Electron microscopy

Grossly dissected neural tubes were fixed with 2% glutaraldehyde in 0.1 M Na pH 7.2 cacodylate buffer 3h at room temperature (RT). Then they were postfixed with 1% osmium tetroxide containing 1.5% potassium cyanoferrate, contrasted with uranyl acetate 2% in water, gradually dehydrated in ethanol (30%-100%) and embedded in Epon. Thin sections (70 nm) of selected zones were collected onto 150 mesh cooper grids, and conterstained with lead citrate before examination with Zeiss EM902 electron microscope operated at 80 kVe (Carl Zeiss-France). Microphotographies were acquired with a charge-coupled device camera (MegaView III) and analysed with ITEM software (Eloïse-France) on MIMA2 Platform.

### Quantification of the number of basal bodies

The number of basal bodies in the floor plate region at the border of the lumen was quantified on transverse sections of E9 and E9.5 FVB/N and *Prnp^-/-^* embryos, after labelling for γTub (≥ 4 embryos analysed per genotype). Signals corresponding to centrioles of cells undergoing mitoses were not counted.

### 1C11 cell cycle analyses

Cells were resuspended at 10^6^ cells/mL in PBS with 0.1% sodium citrate, 50 mg/ml RNaseA, 0.1% Nonidet P40 and 25 mg/ml propidium iodide. The cells were incubated at 4°C for 4 hours before flow cytometric analysis. At least 10,000 cells per sample were analysed. Data were acquired on a FACSCalibur (BD Biosciences) and analysed using the CellQuest software.

### Immunofluorescence microscopy on 1C11 cells

1C11 cells grown on labtek chambers (Nunc) were washed in PBS with 1 mM Ca^2+^ and Mg^2+^ (buffer A) before fixation with 4% formaldehyde in buffer A. Cells were then permeabilised with blocking buffer (buffer A with 20mM Glycine, 1% goat serum and 0.1% Triton X-100) for 15 minutes at room temperature and incubated with Tub^Ac^ and phospho-histone H3 antibodies diluted in buffer A enriched with 1% goat serum and 0.1% Tween for 1 hour at room temperature followed by Alexa Fluor 488-conjugated secondary antibody (Molecular Probes) and DAPI. Immunolabelling was observed with an inverted microscope (Nikon Eclipse TE2000-E) equipped with a black and white CCD CoolSnap HQ2 camera (Photometrics), controlled by NIS-element software. MTT assays analyses were performed on sub-confluent cells as described^2^.

### Number of embryos and litters used for immunoblot analyzes of -α–tubulin variants

The details of the number of embryos and litters analysed (Fig. 5) at each developmental stage are as follows: at E8.25, 5 FVB/N embryos (2 litters with 3 and 2 embryos) and 6 *Prnp^-/-^* embryos (1 litter); at E8.5, 4 FVB/N embryos (2 litters with 1 and 3 embryos) and 3 *Prnp^-/-^* embryos (1 litter); at E9.0, 6 FVB/N embryos (3 litters with 3, 1 and 2 embryos) and 5 *Prnp^-/-^* embryos (3 litters with 1, 1 and 3 embryos); at E9.5, 10 FVB/N embryos (3 litters with 5, 3 and 2 embryos) and 6 *Prnp^-/-^* embryos (4 litters with 1, 1, 1 and 3 embryos); at E10.5, 4 FVB/N embryos (2 litters with 2 embryos per litter) and 4 *Prnp^-/-^* embryos (2 litters with 2 embryos per litter).

References

1 Feraudet, C. *et al.* Screening of 145 anti-PrP monoclonal antibodies for their capacity to inhibit PrPSc replication in infected cells. *The Journal of biological chemistry* **280**, 11247-11258, doi:10.1074/jbc.M407006200 (2005).

2 Pietri, M. *et al.* Overstimulation of PrPC signaling pathways by prion peptide 106-126 causes oxidative injury of bioaminergic neuronal cells. *The Journal of biological chemistry* **281**, 28470-28479, doi:10.1074/jbc.M602774200 (2006).
